# Adaptations of Prefrontal Brain Activity, Executive Functions, and Gait in Healthy Elderly Following Exergame and Balance Training: A Randomized-Controlled Study

**DOI:** 10.3389/fnagi.2016.00278

**Published:** 2016-11-23

**Authors:** Alexandra Schättin, Rendel Arner, Federico Gennaro, Eling D. de Bruin

**Affiliations:** Department of Health Sciences and Technology, Institute of Human Movement Sciences and Sport, ETH ZurichZurich, Switzerland

**Keywords:** elderly, exergame, balance, executive function, gait performance, prefrontal brain activity

## Abstract

During aging, the prefrontal cortex (PFC) undergoes age-dependent neuronal changes influencing cognitive and motor functions. Motor-learning interventions are hypothesized to ameliorate motor and cognitive deficits in older adults. Especially, video game-based physical exercise might have the potential to train motor in combination with cognitive abilities in older adults. The aim of this study was to compare conventional balance training with video game-based physical exercise, a so-called exergame, on the relative power (RP) of electroencephalographic (EEG) frequencies over the PFC, executive function (EF), and gait performance. Twenty-seven participants (mean age 79.2 ± 7.3 years) were randomly assigned to one of two groups. All participants completed 24 trainings including three times a 30 min session/week. The EEG measurements showed that theta RP significantly decreased in favor of the exergame group [*L*_(14)_ = 6.23, *p* = 0.007]. Comparing pre- vs. post-test, EFs improved both within the exergame (working memory: *z* = −2.28, *p* = 0.021; divided attention auditory: *z* = −2.51, *p* = 0.009; divided attention visual: *z* = −2.06, *p* = 0.040; go/no-go: *z* = −2.55, *p* = 0.008; set-shifting: *z* = −2.90, *p* = 0.002) and within the balance group (set-shifting: *z* = −2.04, *p* = 0.042). Moreover, spatio-temporal gait parameters primarily improved within the exergame group under dual-task conditions (speed normal walking: *z* = −2.90, *p* = 0.002; speed fast walking: *z* = −2.97, *p* = 0.001; cadence normal walking: *z* = −2.97, *p* = 0.001; stride length fast walking: *z* = −2.69, *p* = 0.005) and within the balance group under single-task conditions (speed normal walking: *z* = −2.54, *p* = 0.009; speed fast walking: *z* = −1.98, *p* = 0.049; cadence normal walking: *z* = −2.79, *p* = 0.003). These results indicate that exergame training as well as balance training positively influence prefrontal cortex activity and/or function in varying proportion.

## Introduction

The human brain undergoes age-dependent changes that affect motor and cognitive performances of daily living of older adults. The brain aging process is associated with neuroanatomical changes, such as loss of brain tissue and white matter disconnections (Resnick et al., 2003; Raz et al., 2005; Gunning-Dixon et al., 2009), decrease in synthesis and binding of dopamine, serotonin, and acetylcholine (Wang et al., 1995, 1998; Volkow et al., 1998; Bäckman et al., 2006; Schliebs and Arendt, 2011), metabolic changes including mitochondrial dysfunction (Rothman et al., 2011; Hyder and Rothman, 2012; Lin and Rothman, 2014), and changes in neuronal activity pattern (Rossini et al., 2007). Especially the (pre)frontal lobe is vulnerable to age-related degeneration as shown in cross-sectional (Raz et al., 1997; Raz, 2000; Fjell et al., 2009) and longitudinal magnetic resonance imaging studies (Pfefferbaum et al., 2013; Fjell et al., 2014). Prefrontal volume and thickness are associated with executive function (EF) (Yuan and Raz, 2014). EFs are “higher-level” cognitive abilities that control and regulate “lower-level” cognitive processes and goal-directed actions (Alvarez and Emory, 2006; Banich et al., 2009). An interaction of different EF components, e.g., “working memory” (Holtzer et al., 2006), “divided attention” (Sheridan et al., 2003), and “inhibition” (Hausdorff et al., 2005), is needed for gait performance. Reduced gait stability and postural control during dual-task walking suggest that cognitive abilities may affect gait performance (Pichierri et al., 2011; Iosa et al., 2014). Therefore, age-associated reduction of EFs can impact gait and amplify the risk of falling (Mirelman et al., 2012). The assumption is supported by recent work showing that a smaller volume of the prefrontal area is likely to contribute to slower gait speed through slower information processing (Rosano et al., 2012). A greater reliance on cognitive control for motor tasks makes structural differences in the prefrontal cortex interesting from the perspective of age-related decrements in motor control (Seidler et al., 2010). Moreover, disturbances in cortico-cortical and cortico-subcortical connections, e.g., frontal connections with parietal lobe or with basal ganglia, respectively, are classified as higher level gait disorders (Thompson and Nutt, 2007; Scherder et al., 2011).

To improve motor and cognitive performance in elderly, it can be hypothesized that a physical training that also targets EFs might be an important component (Mirelman et al., 2012; Pichierri et al., 2012a). In aging humans, physical exercise can strengthen neuronal structures, synaptic plasticity, and cognitive functions (Cai et al., 2014). Therefore, physical exercise might be a trigger to support molecular and cellular mechanisms for brain plasticity (Kramer et al., 1999; Cotman and Berchtold, 2002). Traditional physical exercise programs of elderly are usually associated with conventional balance exercises. Balance training has been shown to be beneficial for maintenance and improvement of motor performance through changes on subcortical structures (Taube et al., 2008). Balance training performed with the help of video games that incorporate stepping exercises are effective in reducing falls next to improving gait and balance (Okubo et al., 2016). Furthermore, compared to conventional balance training this type of training differently modulates prefrontal brain activity and EFs (Eggenberger et al., 2016). It seems important, in this context, that interventions provide physical activity with decision-making opportunities because these are believed to facilitate the improvement of both motor performance and cognitive function (Yan and Zhou, 2009).

Video game-based training serves as a powerful tool to modulate neural networks and to evaluate underlying neuronal mechanisms (Anguera et al., 2013). Moreover, video games seem to have the potential to train cognitive functions (Zelinski and Reyes, 2009) including reaction time (RT), processing speed, attention, and EFs (Jobe et al., 2001; Kueider et al., 2012). A systematic review concluded that video games are promising for improving cognitive abilities in older adults who have a higher risk of cognitive decline (Kueider et al., 2012). An interactive exergame challenges (divided) attention; participants observe cues on a frontal screen and concurrently execute well-coordinated movements. Furthermore, the combined training of physical and cognitive functions leads to better cognitive function and general functional status in older adults (Law et al., 2014). Two recent reviews, focusing on the interplay between physical function and cognition, concluded that it seems important to combine motor and cognitive exercises into clinical practice to enable older adults to move safer in their physical environment (Segev-Jacubovski et al., 2011; Pichierri et al., 2012a). Especially, computerized interventions seem promising (Green and Bavelier, 2008; Pichierri et al., 2011), in particular, when they consider training principles that enhance (motor) learning (Green and Bavelier, 2008); e.g., direct feedback on performance and rewards for correct responses in the video game-based training scenario.

Despite existing evidence that the older brain shows adaptations associated with cognitive-motor training, the type of training that best promotes these adaptations remains to be elucidated. The aim of this study was, therefore, to compare exergame training with conventional balance training focusing on prefrontal brain activity, EFs, and gait performance. We hypothesized that a combined motor and cognitive training would differently affect prefrontal brain activity, EFs, and spatio-temporal gait parameters when compared to more conventional motor training.

## Materials and methods

### Study design and participants

The study was a randomized controlled trial (RCT) including two parallel running intervention groups. From May through June 2015, potential participants were recruited through public advertisements in local newspapers, on the homepage of the Senior University Zurich (Switzerland), pensioner community ETH Zurich (Switzerland), and in local senior residency dwellings (Zurich, Switzerland). Measurements and interventions were performed in a senior residence dwelling in Zurich (Möhrlistrasse, 8006 Zurich). The intervention started in two blocks, one block started in the middle of June 2015 and the second at the beginning of July 2015. Study measurements were performed before and after the intervention. The ethics committee of the ETH Zurich, Switzerland (EK 2015-N-10) approved the study protocol. The study is registered at Current Controlled Trials under ISRCTN73384012 (http://www.isrctn.com). Before any measurements were performed, all eligible participants had to sign written informed consent according to the Declaration of Helsinki. CONSORT 2010 guidelines were used for the reporting of this parallel-group RCT (Moher et al., 2010).

The potential participants were screened using the Mini Mental State Examination (MMSE) to assess cognitive status and the Geriatric Depression Scale (GDS) to screen for depression. Furthermore, the participants completed a health questionnaire including questions about physical impairments, medical history, anthropometric data, and physical activity level. Participants fulfilling all of the following inclusion criteria were eligible for the study: (1) age ≥ 65 years, (2) live independently or in a senior residency dwelling, (3) non-smoker, (4) healthy (self-reported), (5) able to walk at least 20 m with or without walking aids. Participants were excluded from the study in case they exhibit one of the following exclusion criteria: (1) mobility impairments, (2) severe health problems (e.g., recent cardiac infarction, uncontrolled diabetes, or uncontrolled hypertension), (3) orthopedic or neurological diseases that prevent training participation, (4) Alzheimer disease or dementia, (5) rapidly progressive or terminal illness, (6) acute or chronic illness, (7) history of stroke, (8) history of dizziness or individuals with a recent head injury, (9) medications that act on neuronal level (e.g., Psychotropic medications), (10) cognitive impairments (MMSE <22 points), (11) signs of an upcoming depression.

The intended study size of 30 to 40 participants was based on similar training studies (Pichierri et al., 2012a,b). For study group randomization, a randomization program was used (www.randomization.com). With the specification of two intervention groups and the intended number of participants, the program automatically generated a list including exergame and balance training in a randomized and balanced fashion and a block size of two. The participant assignment started at one and was hence continuously filled-up according to the chronological measurement protocol. The random allocation sequence was performed by a study investigator while the participant enrollment and assignment was done by another study investigator. Blinding of the supervisors was not possible, because they performed the measurements and the intervention. Participants were blinded to the expected study outcome.

### Training interventions

In the period from June 2015 to September 2015, the participants performed 30 min sessions three times per week on separate days. The trainings were scheduled individually from Monday to Friday with certain regularity for each week and a guideline of no more than one training per day. The 24 training sessions were distributed within a period of 8 to 10 weeks, while a maximum of 2 weeks holiday interruption was allowed. Each training session included a warm-up (5 min), a training (20 min), and a cool-down phase (5 min). Three postgraduate students instructed the participants and ensured that training principles of progression and overload were present in both groups (Ammann et al., 2014). Training intensity was individually adapted to achieve a moderate to vigorous training level (Chodzko-Zajko, 2014). The time frame and training intensity was based on studies illustrating positive training effects in older adults performing a video game on a dance plate (Pichierri et al., 2012a; Law et al., 2014; Eggenberger et al., 2016) and on current recommendations for achieving physical fitness and fall prevention in elderly (Chodzko-Zajko, 2014). The participants trained in groups to enhance motivation and motor learning (Law et al., 2014).

#### Exergame training

The exergame group performed a cognitive-motor training including an interactive video game-based physical exercise. On a pressure sensitive plate (Impact Dance Platform, 87.5 × 87.5 × 2.5 cm, Positive Gaming BV, BZ Haarlem, Nederland) the participants performed specific whole body movements driven by a video game presented on a frontal screen. Electronic sensors, on the platform, detected position and timing information that were then used to provide participants with real-time visual feedback. Through foot pushes on the plate arrows (right, left, top, and bottom), the participants interacted in the gaming interface. The dance platform was connected by USB to a desktop computer and to a beamer projecting the video game on a wall.

The exergame allowed the implementation of training principles as previously described (Healy et al., 2014); a feedback system to facilitate training, offering individual difficulty zones to catch each participant's training level, individually adjusted task difficulty to facilitate retention and offering variability of training to enhance task transfer. The participants played four different games developed and provided by dividat (Schindellegi, Switzerland) (Table 1). The games specifically train EFs that are (1) associated with the prefrontal brain area (Yuan and Raz, 2014), (2) are important for the control and regulation of “lower-level” cognitive processes and goal-directed actions (Alvarez and Emory, 2006; Banich et al., 2009), and (3) can impact on gait and amplify the risk of falling (Mirelman et al., 2012). Each game included different difficulty levels to ensure adaption and progression to the abilities of each individual. Furthermore, each game was accentuated with music. One session included six to seven rounds while one round of a game lasted about 3 min. The intervention started with “Balloon” and “Step” and was extended with “Space” and “Season” in case “Balloon” and “Step” were mastered by an individual during the intervention period. The participants trained 18–21 min including individual break sessions.

**Table 1:** **Video game description**.

**Video game**	**Task**
Balloon	If a balloon meets one of the four circles, the participant has to push the corresponding arrow on the plate. The balloons come from random sides of the game play screen and move toward one of the four circles. The balloons may exhibit different colors and the participant has to inhibit moving, in case the balloon exhibits the “wrong” color.
Step	If an object meets one of four target object points (corresponding to front, back, left, and right arrows), the participant has to push the relevant arrow direction on the plate. The objects appear in the middle of the play screen and run to one of the four target objects. The participant has to inhibit moving when the shape of the appearing objects (e.g., triangles) is incompatible with the four target objects (e.g., squares).
Season	Participants view four different scenes (four seasons). Each of the four scenes corresponds to one arrow direction of the plate. If within a scene a moving animation (e.g., bird) appears, the participant has to push the corresponding arrow on the plate as quickly as possible.
Space	The game is based on the rules of the tile-matching video game Tetris. The participant arranges the tile objects using the relevant arrow directions on the plate.

#### Balance training

The balance group trained conventional balance training. Participants performed repetitive static and dynamic exercises on stable and unstable surfaces to challenge their balance. To ensure a progression in difficulty levels, the exercises were increased in difficulty by requiring exercise performance in either bipedal or monopedal stance positions, by opening or closing the eyes, and through causing external perturbations or using a moving base of support.

### Measurement of spatio-temporal gait parameters

Temporal and spatial gait parameters were measured with the Physilog (Gait up Sàrl, Lausanne, Switzerland) via wearable standalone movement sensors (50 × 37 × 9.2 mm, 19 grams, anatomical curved shape) containing inertial sensors. The focus of this study was on gait speed, cadence, and stride length. The validity of the Physilog has been previously established (Aminian et al., 1999; Dubost et al., 2006; de Bruin et al., 2007). At the right and left forefoot, the sensors were fixed with elastic straps for flat over ground gait analysis. A button on the sensors was used for the start and stop of measurements. For further analysis, a micro-USB port allowed data transfer to the computer.

Over a 10 m walkway, participants performed a single-task walking condition (both preferred and fast gait speed) and a dual-task walking condition, i.e., preferred and fast gait speed whilst counting backwards in steps of seven. Application of the dual-task paradigm aims to quantify the automaticity of movement (Abernethy, 1988; Wright and Kemp, 1992; Wulf et al., 2001). For the single-task, participants were instructed to position themselves at the beginning of the walkway and were asked to walk with their comfortable self-paced (or fast) speed. For the dual-task, the participants got a random starting number from which they had to count backwards in steps of seven while walking with their comfortable self-paced (or fast) speed. The instructions were as follows: (1) “Walk with your comfortable preferred/fast speed right to the end of the walkway.” (2) “Walk with your comfortable preferred/fast speed right to the end of the walkway counting backwards from [223, 238, and 245].” The participants had to count loud and regularly; otherwise, the trial was recorded as failure. The participants performed the following procedure: (1) self-paced single-task walking, (2) self-paced dual-task walking, (3) fast single-task walking, and (4) fast dual-task walking. Each tested condition was repeated three successful times to obtain representative samples. The means out of the three successful runs and from the left and right foot were used for further data analysis. To analyze steady state walking, acceleration, and deceleration steps were removed from the data and not counted.

### Measurement of executive functions

Participants performed four tests from the test battery Test for Attentional Performance (TAP) (PSYTEST, Psychologische Testsysteme, Herzogenrath) testing different forms of EFs. The selected tests are geared to the component factors underlying EFs: Inhibition and switching, working memory, and (selective) attention (Alvarez and Emory, 2006; Grady, 2012). The TAP is valid as the subtests measure different and statistically independent attentional and executive aspects (Zimmermann and Fimm, 2002). On a personal computer, the participants performed the following test procedure: (1) *working memory (5 min)*: The software presented two-digit numbers. The participant compared presented numbers with previously exposed numbers and had to push the button when the presented number was the same as the penultimate number; (2) *set-shifting/flexibility task (minimum 1 min and 45 s)*: The software presented a letter and a number—one on the left and one on the right side of the computer screen in a randomized order. The participant had to react on the target stimulus (e.g., “letter”—“number”—“letter”…) pushing the right (appearance on the right side) or left (appearance on the left side) button. The button press triggered the presentation of the next stimulus; (3) *divided attention (3 min 25 s)*: The software concurrently presented visual and acoustic signals. In a 4 × 4 matrix, the visual task consisted of crosses appearing in a random configuration. The participant had to push the button when the crosses formed the corners of a square. The acoustic part consisted of low and high beeps playing in a randomized sequence. The participant had to push the button when a sequence of two similar tones appeared, e.g., low-low or high-high, respectively; (4) *go/no-go/inhibition task (2 min)*: The software randomly presented stimulus (x) or (+). The participant had to push the button in the presence of the key stimulus (x) and had to inhibit the push in the presence of the non-key stimulus (+). For each test, the program stored the RT. To avoid learning effects each test was preceded by a short pre-test version to familiarize with the test.

### Measurement of prefrontal brain activity

#### Electroencephalography procedure

A portable electroencephalography (EEG) device (*HeadCoach*™, Alpha-Active Ltd, Devon, UK) was used in order to record scalp electro-voltage activity (sampling frequency: 128 Hz, band-pass hardware filter: 1–32 Hz) from two active (positive inputs) electrodes (Ambu® White Sensor, Ambu A/S, Denmark), placed on Fp1 and Fp2 according to the 10–20 EEG system (Jasper, 1958). Three additional electrodes were used: A passive Driven-Right-Leg (DRL) reference electrode (Jasper, 1958) and two active (negative inputs) Common Mode Sense (CMS) electrodes, placed at Fpz and M1/M2 (mastoid process), respectively. DRL and CMS electrodes were used in order to reduce participant's electromagnetic interference and to improve the common-mode rejection ratio of the two recording positive inputs channels. Before electrode placement, the skin was prepared with an abrasive skin preparation gel (Nuprep™, Weaver and Company, Aurora, USA) and then cleaned with alcohol-free wipe.

The brain signals acquisition was carried out during the divided attention task of the TAP that included the simultaneous presentation of visual and auditory stimuli (see description in paragraph measurement of executive functions). EEG measurements were time-locked to both auditory and visual stimuli of the correct responses (visual: 17 correct responses out of 100 total stimuli; auditory: 16 correct responses out of 200 total stimuli). EEG analysis was triggered with the stimulus onset from the cognitive test, in both auditory and visual stimuli, using the following naïve approach: (1) the onset of the first auditory stimulus was determined and recorded using the .wav file (produced by the EEG software and chronological to the EEG data file), (2) the onset of the first visual stimulus, as well as all the subsequent auditory and visual stimuli were determined calculating the time lasting from the first auditory stimuli and the defined time intervals between the onset of either auditory and visual stimuli (as defined by the TAP software).

Moreover, the protocol included the measurement of brain signals during gait performance. The measurements were performed concurrent with spatio-temporal gait measurements (see description in paragraph measurement of spatio-temporal gait parameters). The EEG measurement was controlled by pushing the start button when participants started to walk and by pushing the stop button when participants stopped to walk. For each run and condition, the same EEG procedure was performed. To enable the measurement during walking, an extension cable was used to connect the EEG system with the personal computer.

#### Data processing

Off-line signal processing was performed using a custom written script and both EEGLAB (Delorme and Makeig, 2004) and ERPLAB (Lopez-Calderon and Luck, 2014) toolboxes for MATLAB (Mathworks, Natick, MA). To facilitate further EEG analysis, raw signals were first resampled to 100 Hz, and subsequently time-locked to either auditory or visual stimuli in 4 s epochs (–1500 ms pre- and 2500 ms post-stimulus onset), which were analyzed separately. Then, EEG epochs containing artifacts (e.g., ocular movements and eye blinks) were automatically rejected using an absolute voltage threshold criterion (epochs rejected when peaks amplitude ≥ ± 200 μV).

EEG spectral power was calculated through Power Spectrum Density (PSD) extraction, using the pwelch method (Welch, 1967) implemented in the DK_PSD function (Dickter and Kieffaber, 2013). The following frequency bands were determined a priori and then extracted: Total bandwidth (1–30 Hz), delta (1–3.5 Hz), lower theta (3.5–5.5 Hz), upper theta (5.5–7.5 Hz), lower alpha (7.5–10 Hz), upper alpha (10–12.5 Hz), and beta (12.5–30 Hz). All the further EEG analysis was performed clustering Fp1 and Fp2 channels. RP density was computed for each frequency band in each participant as previously described (Moretti et al., 2013; Moretti, 2015), using the following equation:

$$\begin{array}{l} \text{RP}\left(\lambda \right)=\;\frac{\text{AP}\left(\lambda \right)}{\text{MP}\left(\text{TB}\right)} \end{array}$$

where RP = relative power, λ = frequency band, AP = absolute power peak, MP = mean power spectra, TB = total bandwidth). RP values were then natural-log normalized. The analysis of event-related potentials was not performed because of the naïve triggering approach and the use of a low-density EEG device.

### Fear of falling, cognitive status, and depression

The short falls efficacy scale international (FES-I) was used to measure concern about falling through the combination of phrases and matching pictures. The scale assesses both easy and difficult physical and social activities and contains seven items with a 4-point scale (1 = not at all concerned, 2 = somewhat concerned, 3 = fairly concerned, 4 = very concerned). The short FES-I is a feasible scale to assess fear of falling in elderly (Kempen et al., 2008).

The cognitive status was determined using the MMSE, a reliable and valid test to quantitatively estimate severity of cognitive impairments (Folstein et al., 1975; Tombaugh and McIntyre, 1992). The test has a maximal score of 30 points and is categorized into seven categories: (1) orientation to time, (2) orientation to place, (3) registration of three words, (4) attention and calculation, (5) recall of three words, (6) language, and (7) visual construction.

The GDS, for valid and reliable depression screening (Yesavage et al., 1983), was used to identify depression status in older adults. The short form has 15 questions focusing on worries of the individual, and the way they conceive and interpret their quality of life (Yesavage and Sheikh, 1986). The self-report questionnaire can be answered with yes/no responses.

### Statistical analysis

Statistical Package for Social Sciences 22.0 for Windows (SPSS, Inc; Chicago, Illinois) was used for all statistical analyses. A per protocol analysis was performed. Normality of the sample was tested by Shapiro-Wilk. Given that the assumption of normality was not met, the data were rank-ordered in order to perform, for each variable, a two-way repeated measures analysis of variance (ANOVA) with one within-subjects factor (time: Pre-test–post-test) and one between-subjects factor (intervention group: Balance/exergame). This allows to compare main effects of time and time × group interaction effects, using the Puri and Sen L Statistics for ranked data (Thomas et al., 1999). L value was calculated using Pillai's Trace. *Post-Hoc* analyses to determine differences in time (within groups) were performed using the Wilcoxon Signed Rank Test. Baseline (pre-test) comparisons were undertaken using Mann-Whitney *U*-test. A *p*-value ≤ 0.05 was considered significant. Effect sizes assessing meaningfulness of differences within group design were calculated and expressed using the following equation:

$$\begin{array}{l} \text{r}=\;\frac{\text{Z}}{\sqrt{N}} \end{array}$$

where Z = Z-score and N = amount of participants.

An effect size of *r* = 0.1 is considered a “small” effect, around 0.3 a “medium” effect, and 0.5 and above a “large” effect. Effects sizes assessing differences between × within group design were calculated and expressed as $\eta_{G}^{2}$ where percentage of the total variance can be accounted for by group membership (Lakens, 2013), using the following equation:

$$\begin{array}{l} \eta_{G}^{2}=\;\frac{SS_{effect}}{SS_{effect}+SS_{error}+SS_{error\;within}} \end{array}$$

where SS = sum of squares.

## Results

**Figure 1:**
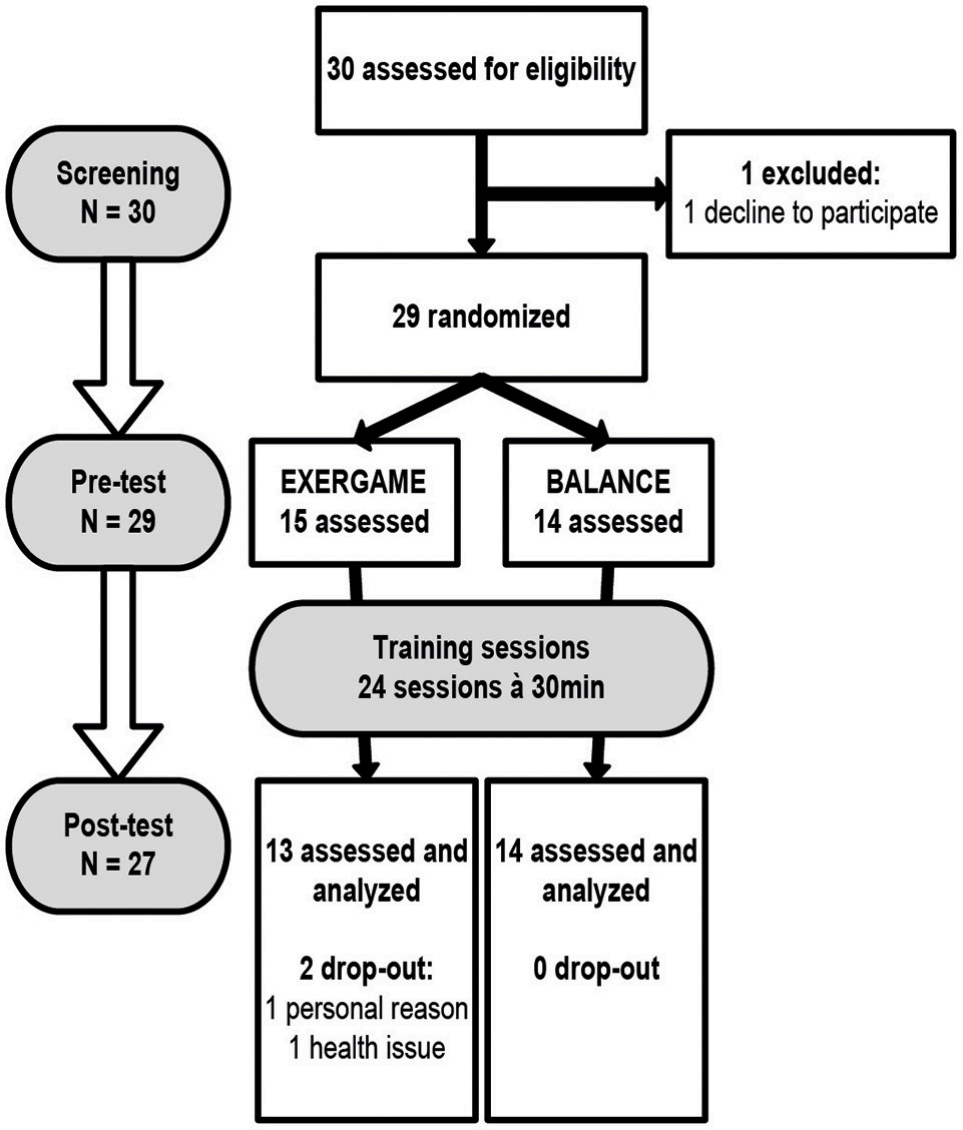
**Study flow chart**. Participants were randomly assigned to either EXERGAME or BALANCE group. Both groups trained 24 times within a period of 8 to 10 weeks. The screening included a health questionnaire, geriatric depression scale, mini mental status examination, and short falls efficacy scale international. Prefrontal brain activity, executive functions, and spatio-temporal gait parameters were assessed before and after the intervention.

A total of 29 older adults were randomized into the two groups: exergame and balance training. 27 participants completed the whole training procedure with each participant completing all 24 scheduled training sessions. The study flow chart is presented in Figure 1. The analysis does not consider intention-to-treat analysis because of a clear description of the reason(s) for dropout [CONSORT 2010 guidelines Moher et al., 2010]. Two participants were excluded from analysis for not completing the study. Dropout reasons (one personal and one health issue) were not associated with the intervention. Table 2 summarizes demographic data of the participants.

**Table 2:** **Demographic characteristics and screening values**.

	**Exergame group**	**Balance group**	
Gender (female/male)	5/8	7/7	
			***p* (two-tailed)**
Age (years)	80 (73; 83)	80 (72.25; 81.75)	0.685
Mini mental status examination	29 (29; 30)	28.5 (27; 29)	0.259
Geriatric depression scale	1 (0; 2)	2.5 (1; 4.75)	0.085
Short falls efficacy scale international	7 (7; 8)	8.5 (7; 10)	0.076

*Data are number of participants or median (interquartile range) values as indicated. p ≤ 0.05*.

### Prefrontal brain activity

For the analysis, some participants had to be excluded due to technical problems (the included number of participants is illustrated in Tables 3, 4). The total amount of epochs, used for analysis, were for the exergame group pre (*N* = 147), post (*N* = 161) and for the balance training group pre (*N* = 107), post (*N* = 122). In every participant, eight epochs had to be excluded because epoch time windows overlapped. Table 3 summarizes the interaction effects for RP values separately for both auditory and visual stimuli. Significant time × group interaction effect was present for theta RP (auditory stimuli) [*L*_(14)_ = 6.230, *p* = 0.007]. The results of the pre- vs. post-tests comparisons are presented in Table 4. The exergame group significantly decreased theta RP (auditory stimuli) (*z* = −2.37, *p* = 0.016). The naïve triggering modalities and the low-density EEG device allowed no analysis of the data during gait performance.

**Table 3:** **Interaction effects (time × intervention) of repeated measures Puri & Sen-analyses of ranked data for relative power values**.

	**Pillai's trace *r^2^***	***L* = (*N* − 1)*r*^2^**	***p***	**$\eta_{G}^{2}$**
**AUDITORY STIMULI**				
Delta	0.218	3.052	0.079	0.01
Theta	0.445	6.230	0.007^**^	0.02
Alpha low	0.216	3.024	0.081	0.08
Alpha high	0.223	3.122	0.076	0.00
Beta	0.125	1.750	0.195	0.00
**VISUAL STIMULI**				
Delta	0.020	0.280	0.618	0.00
Theta	0.215	3.010	0.082	0.00
Alpha low	0.260	3.640	0.052	0.01
Alpha high	0.174	2.436	0.122	0.00
Beta	0.240	3.360	0.064	0.01

N = 15; exergame group N = 7 and balance group N = 8. ^*^p ≤ 0.05,

** *p ≤ 0.01, p-values are one-tailed and based on normalized data. $\eta_{G}^{\text{2}}$, effect size. Bold values indicate significance and medium or large effect size*.

**Table 4:** **Pre- vs. post-test of relative power values for exergame and balance group**.

**Exergame group**	**Pre (*N* = 8)**	**Post (*N* = 9)**	***z***	***p***	***r***
**AUDITORY STIMULI**					
Delta	1.79 (1.61; 2.07)	1.59 (1.33; 1.95)	−1.52	0.156	**−0.37^a^**
Theta	1.86 (1.55; 2.42)	1.70 (1.24; 1.86)	−2.37	0.016^*^	**−0.57^b^**
Alpha low	1.24 (1.16; 1.38)	1.00 (0.80; 1.35)	−1.18	0.297	−0.29
Alpha high	1.14 (0.67; 1.38)	0.83 (0.55; 1.16)	−1.18	0.297	−0.29
Beta	−0.21(−0.6; 10.06)	−0.19 (−0.44; 0.14)	−1.18	0.297	−0.29
**VISUAL STIMULI**					
Delta	−0.08 (−0.63; 0.07)	−0.22 (−0.46; 0.14)	−1.01	0.375	−0.25
Theta	−0.82 (−1.29; −0.38)	−0.60 (−0.96; −0.28)	−1.18	0.297	−0.29
Alpha low	−0.57 (−1.23; −0.21)	−0.32 (−0.78; 0.02)	−1.70	0.109	**−0.41^a^**
Alpha high	−0.77 (−1.14; −0.09)	−0.44 (−1.14; −0.09)	−0.85	0.469	−0.21
Beta	−0.70 (−0.90; −0.74)	−0.31 (−0.74; −0.04)	−1.52	0.078	**−0.37^a^**
**Balance group**	**Pre (*N* = 10)**	**Post (*N* = 9)**			
**AUDITORY STIMULI**					
Delta	1.70 (1.28; 2.10)	1.99 (1.55; 2.29)	−0.56	0.641	−0.13
Theta	1.81 (1.63; 2.02)	1.92 (1.65; 2.10)	−0.70	0.547	−0.16
Alpha low	0.79 (0.44; 1.17)	1.30 (0.69; 1.47)	−1.54	0.148	**−0.35^a^**
Alpha high	0.79 (0.29; 1.28)	1.26 (0.77; 1.46)	−1.82	0.078	**−0.42^a^**
Beta	−0.16 (−0.33; 0.20)	−0.26 (−0.90; 0.21)	−1.54	0.148	**−0.35^a^**
**VISUAL STIMULI**					
Delta	−0.12 (−0.48; 0.18)	−0.14 (−0.68; 0.15)	0.00	1.000	0.00
Theta	−0.24 (−1.49; 0.03)	−0.77 (−1.55; −0.43)	−1.40	0.195	**−0.32^a^**
Alpha low	−0.27 (−1.12; −0.13)	−0.60 (−0.90; −0.37)	−0.70	0.547	−0.16
Alpha high	−0.21 (−1.81; −0.07)	−0.97(−1.74; −0.32)	−1.12	0.313	−0.26
Beta	−0.35 (−1.38; 0.07)	−0.84 (−1.16; −0.28)	−0.70	0.547	−0.16

*Normalized data from pre-post are median values (interquartile range) as indicated*.

* *Significant within-group differences pre-post (p ≤ 0.05) calculated with Wilcoxon signed-rank test (p-values are two-tailed). For effect size r, r = 0.1 − 0.29 indicates a small effect*,

a r = 0.3 − 0.49 indicates a medium effect, and

b *r ≥ 0.5 indicates a large effect. Bold values indicate significance and medium or large effect size*.

### Executive functions

For the analysis, some participants had to be excluded because of technical problems (the included number of participants is illustrated in Tables 5, 6). No significant interactions were present for any of the TAP tests (Table 5). The results of the pre- vs. post-tests comparisons are presented in Table 6. Comparing pre- vs. post-test for the exergame group, all TAP tests showed a significant decrease of the RT (working memory: *z* = −2.28, *p* = 0.021; divided attention auditory: *z* = −2.51, *p* = 0.009; divided attention visual: *z* = −2.06, *p* = 0.040; go/no-go: *z* = −2.55, *p* = 0.008; set-shifting: *z* = −2.90, *p* = 0.002). The balance group showed a significant decrease of the RT for the set-shifting test (*z* = −2.04, *p* = 0.042).

**Table 5:** **Interaction effects (time × intervention) of repeated measures Puri & Sen-analyses of ranked data for Test for Attentional Performance**.

	**Pillai's trace *r^2^***	***L* = (*N* − 1)*r*^2^**	***p***	**$\eta_{G}^{2}$**
Working memory^+^	0.144	3.456	0.061	0.00
Divided attention auditory^++^	0.040	1.000	0.326	0.09
Divided attention visual	0.043	1.118	0.297	0.02
Go/No-go	0.063	1.701	0.206	0.11
Set-shifting	0.023	0.598	0.446	0.04

*Test for Attentional Performance measured reaction time [ms]. N = 27; exergame group N = 13 and balance group N = 14*.

+ N = 25; exergame group N = 12 and balance group N = 13;

++ *N = 26; exergame group N = 12 and balance group N = 14. p ≤ 0.05, p-values are one-tailed. $\eta_{G}^{2}$, effect size*.

**Table 6:** **Pre- vs. post-test of Test for Attentional Performance for exergame and balance group**.

**Exergame group**	**Pre (*N* = 13)**	**Post (*N* = 13)**	***z***	***p***	***r***
Working memory	876.5 (701.8; 1104.5)^+^	750 (649.5; 907.5)	−2.28	0.021^*^	**−0.46^a^**
Divided attention auditory	631.5 (580; 756.25)^++^	598 (533.5; 629)	−2.51	0.009^**^	**−0.50^b^**
Divided attention visual	1109 (835; 1291)	945 (879.5; 1082)	−2.06	0.040^*^	**−0.40^a^**
Go/No-go	450 (426; 496)	421 (410.5; 451.5)	−2.55	0.008^**^	**−0.50^b^**
Set-shifting	1723 (1143.5; 2273.5)	1405 (855; 1727.5)	−2.90	0.002^**^	**−0.57^b^**
**Balance group**	**Pre (*N* = 14)**	**Post (*N* = 14)**			
Working memory	802 (682.3; 871.5)	767 (726; 926.5)^+++^	−0.31	0.787	−0.06
Divided attention auditory	720 (606.25; 787.75)	665.5 (604.25; 733.5)	−1.10	0.296	−0.21
Divided attention visual	961 (841.75; 1082.25)	907.5 (855.5; 1054.25)	−0.60	0.583	−0.11
Go/No-go	472 (448.5; 503.75)	466 (417.3; 509)	−0.25	0.802	−0.05
Set-shifting	1196 (1045.5; 1752.25)	1110 (984; 1409.5)	−2.04	0.042^*^	**−0.39^a^**

The table presents the reaction time [ms] for each test. Data from pre-post are median values (interquartile range) as indicated. *Significant within-group differences pre-post (

* p ≤ 0.05 and

** *p ≤ 0.01) calculated with Wilcoxon signed-rank test (p-values are two-tailed). For effect size r, r = 0.1 − 0.29 indicates a small effect*,

a r = 0.3 − 0.49 indicates a medium effect, and

b *r ≥ 0.5 indicates a large effect*.

+ N = 12;

++ N = 12;

+++ *N = 13. Bold values indicate significance and medium or large effect size*.

### Spatio-temporal gait parameters

No significant interactions were present for any of the gait parameters (Table 7). The results of the pre- vs. post-tests comparisons are presented in Table 8. Comparing pre- vs. post-test, the exergame group significantly improved gait speed during dual-task walking at preferred (*z* = −2.90, *p* = 0.002) and at fast (*z* = −2.97, *p* = 0.001) speed. Furthermore, cadence was significantly enhanced for dual-task walking at preferred speed (*z* = −2.97, *p* = 0.001) and stride length was significantly improved for dual-task walking at fast speed (*z* = −2.69, *p* = 0.005). Comparing pre- vs. post-test, the balance group significantly improved gait speed for single-task walking at preferred (*z* = −2.54, *p* = 0.009) and at fast (*z* = −1.98, *p* = 0.049) speed and for dual task walking at fast speed (*z* = −1.98, *p* = 0.049). Moreover, gait cadence was significantly improved for single-task walking at preferred speed (*z* = −2.79, *p* = 0.003).

**Table 7:** **Interaction effects (time × intervention) of repeated measures Puri & Sen-analyses of ranked data for spatio-temporal gait parameters**.

**Time × intervention interaction**	**Pillai's trace *r^2^***	***L* = (*N* − 1)*r*^2^**	**p**	**$\eta_{G}^{2}$**
**SPEED [M/S]**				
Single-task normal	0.001	0.026	0.880	0.01
Single-task fast	0.068	1.768	0.189	0.00
Dual-task normal	0.001	0.026	0.882	0.01
Dual-task fast	0.004	0.104	0.753	0.00
**CADENCE [STEPS/MIN]**				
Single-task normal	0.003	0.078	0.774	0.05
Single-task fast	0.108	2.808	0.094	0.08
Dual-task normal	0.032	0.832	0.371	0.00
Dual-task fast	0.031	0.806	0.376	0.01
**STRIDE LENGTH [M]**				
Single-task normal	0.000	0.000	0.915	0.00
Single-task fast	0.007	0.182	0.682	0.01
Dual-task normal	0.014	0.364	0.554	0.01
Dual-task fast	0.070	1.820	0.183	0.01

*N = 27; exergame group N = 13 and balance group N = 14. p ≤ 0.05, p-values are one-tailed. $\eta_{G}^{2}$, effect size, normal: preferred speed, fast: fast speed*.

**Table 8:** **Pre- vs. post-test of spatio-temporal gait parameters for exergame and balance group**.

**Exergame group**	**Pre (*N* = 13)**	**Post (*N* = 13)**	***z***	***p***	***r***
**SPEED** [M/S]					
Single-task normal	1.13 (0.92; 1.22)	1.19 (1.06; 1.29)	−1.64	0.110	**−0.32^a^**
Single-task fast	1.54 (1.31; 1.63)	1.49 (1.38; 1.64)	−1.29	0.216	−0.25
Dual-task normal	1.02 (0.88; 1.18)	1.08 (0.95; 1.29)	−2.90	0.002^**^	**−0.57^b^**
Dual-task fast	1.40 (1.13; 1.47)	1.45 (1.10; 1.62)	−2.97	0.001^**^	**−0.58^b^**
**CADENCE [STEPS/MIN]**					
Single-task normal	103.7 (95.9; 109.3)	106.3 (100.9; 113.3)	−1.78	0.080	**−0.35^a^**
Single-task fast	129.7 (114.9; 142.6)	125.6 (118.48; 134.0)	−0.45	0.685	−0.09
Dual-task normal	104.4 (94.37; 107.3)	104.1 (96.8; 116.7)	−2.97	0.001^**^	**−0.58^b^**
Dual-task fast	116.7 (106.6; 129.8)	124.2 (110.3; 131.1)	−1.43	0.168	−0.28
**STRIDE LENGTH [M]**					
Single-task normal	1.26 (1.14; 1.34)	1.30 (1.26; 1.37)	−1.92	0.057	**−0.38^a^**
Single-task fast	1.39 (1.35; 1.48)	1.43 (1.37; 1.48)	−1.15	0.273	−0.23
Dual-task normal	1.18 (1.10; 1.30)	1.21 (1.15; 1.32)	−1.57	0.127	**−0.31^a^**
Dual-task fast	1.33 (1.21; 1.39)	1.36 (1.29; 1.53)	−2.69	0.005^**^	**−0.53^b^**
**Balance group**	**Pre (*N* = 14)**	**Post (*N* = 14)**			
**SPEED [M/S]**					
Single-task normal	1.06 (0.92; 1.25)	1.12 (1.04; 1.41)	−2.54	0.009^**^	**−0.48^b^**
Single-task fast	1.44 (1.29; 1.62)	1.56 (1.40; 1.78)	−1.98	0.049^*^	**−0.37^a^**
Dual-task normal	1.00 (0.87; 1.05)	1.04 (0.90; 1.24)	−1.54	0.135	−0.29
Dual-task fast	1.22 (1.16; 1.47)	1.40 (1.17; 1.54)	−1.98	0.049^*^	**−0.37^a^**
**CADENCE [STEPS/MIN]**					
Single-task normal	108.2 (100.1; 112.8)	115.1 (101.1; 116.9)	−2.79	0.003^**^	**−0.53^b^**
Single-task fast	133.1 (117.78; 147.4)	137.4 (129.6; 150.2)	−1.92	0.058	**−0.36^a^**
Dual-task normal	101.9 (92.0; 111.8)	111.1 (99.8; 115.4)	−1.85	0.068	**−0.35^a^**
Dual-task fast	115.9 (110.4; 131.7)	127.0 (117.5; 135.0)	−1.35	0.194	−0.26
**STRIDE LENGTH [M]**					
Single-task normal	1.18 (1.06; 1.34)	1.29 (1.04; 1.39)	−1.92	0.058	**−0.36^a^**
Single-task fast	1.32 (1.11; 1.53)	1.40 (1.15; 1.56)	−0.91	0.391	−0.17
Dual-task normal	1.17 (1.02; 1.28)	1.20 (0.97; 1.34)	−0.79	0.463	−0.15
Dual-task fast	1.30 (0.93; 1.53)	1.37 (1.07; 1.42)	−1.10	0.296	−0.21

Data from pre-post are median values (interquartile range) as indicated. *Significant within-group differences pre-post (

* p ≤ 0.05 and

** *p ≤ 0.01) calculated with Wilcoxon signed-rank test (p-values are two-tailed). For effect size r, r = 0.1 − 0.29 indicates a small effect*,

a r = 0.3 − 0.49 indicates a medium effect, and

b *r ≥ 0.5 indicates a large effect. Normal: preferred speed, fast: fast speed. Bold values indicate significance and medium or large effect size*.

## Discussion

This study compared exergame training with conventional balance training focusing on prefrontal brain activity (RP), EFs, and gait performance. We hypothesized that a combined motor and cognitive training would differently affect prefrontal brain activity, EFs, and spatio-temporal gait parameters when compared to more conventional motor training. The results of the brain activity measurement showed that theta RP (auditory stimuli) significantly decreased post-intervention in favor of the exergame group. Comparing pre- vs. post-test, four EFs improved within the exergame group (working memory, divided attention, go-/no-go, and set-shifting) and one within the balance group (set-shifting). In addition, spatio-temporal gait parameters primarily improved within the exergame group under dual-task conditions and within the balance group under single-task conditions, respectively.

### Prefrontal brain activity

The results showed that theta RP (auditory stimuli) significantly decreased in the exergame group. This decrease in theta power band frequency contrasts the behavior we might expect due to the aging process; this parameter is rather expected to increase with age (Rossini et al., 2007). Furthermore, previous research findings suggest that an increased frontal theta power reflects an aging mechanism in which slow cortical waves (e.g., theta oscillations) move from a stable state (lower EEG power) to a relatively unstable state (higher EEG power) (Ho et al., 2012), a mechanism called age-related “slowing” (Rossini et al., 2007). The results of our study, therefore, might mean that exergame training is able to ameliorate age-related “slowing” of EEG that is, in turn, linked to superior cognitive functioning, improved motor performance, and enhanced sensory processing (Rossini et al., 2007). However, we should bear in mind that the findings on the slowing effect are not consistently reported (Rossini et al., 2007), since there are also studies reporting that no increase in theta power can be observed in older adults (Cummins and Finnigan, 2007), or spontaneous delta power is higher in young compared to healthy older adults (Gaál and Czigler, 2015).

According to the de-differentiation view, an increase of theta RP frequency reflects a difficulty in recruiting specialized neural mechanisms (Cabeza, 2002). One might speculate that the exergame training helped to increase connections between different brain areas, because theta RP decreased, thus enabling better control and eased recruitment of specialized neural mechanisms (Cabeza, 2001; Ho et al., 2012). However, these relations can be only hypothesized so far, because it still remains unclear how age-related changes affect EEG (slow) oscillations and whether they seem to mediate control processes. It might well be that reduced frontal theta RP reflects an executive impediment in top-down control due to increasing task demands (Gajewski and Falkenstein, 2014), instead of being due to an increase in brain connectivity. Furthermore, theta RP linked to the auditory stimuli might also be enhanced because of working memory improvements by exergame training. Perceptual operations of acoustic inputs are encoded by working memory and these processes are affected by age-limited processing resources (Wingfield et al., 2005). Considering the exergame group, it might be that working memory improved to such an extent that an elevation of the processing resources for acoustic inputs was achieved. Our results warrant further research in the functional and structural changes of the brain caused by exergaming.

### Executive functions

No time × group interaction effects were present for any of the measured EFs. The exergame group, however, significantly improved in four measures, while for the balance group only set-shifting improved. A previous study investigating exergame and balance training in elderly and measuring EFs, especially shifting, working memory, and inhibition, came to a similar result (Eggenberger et al., 2016).

Video games serve as a powerful tool to enhance cognitive abilities in elderly (Anguera et al., 2013). On the other hand, a review concluded that computerized cognitive training leads to small to moderate improvements in certain cognitive domains with no effects on EFs (Ballesteros et al., 2015). We assume that the video game-based training efficacy might be ensured by video game characteristics that are close to the cognitive outcome of interest (Lampit et al., 2014). A study concluded that different genres of video games may not have equal positive effects on the same cognitive aspect (Dobrowolski et al., 2015). The current video games included cues that specifically trained divided attention, inhibition (go/no-go), set-shifting, and working memory. For example, cognitive flexibility might be trained by video games that train manipulation of multiple information sources (Glass et al., 2013). Furthermore, the outcomes should be considered with caution since different cognitive abilities were defined as EF components measured with different kinds of EF assessments. Finally, the combination of physical exercise and cognitive training leads to improvement of general cognitive functions and memory in older adults (Law et al., 2014). Thus, physical exercise might be executed in a cognitively challenging environment to effectively induce cognitive benefits (Fabel and Kempermann, 2008; Fabel et al., 2009). Exergames are a specific concurrent combination of cognitive and motor training including a video game-based physical exercise. Our interactive exergame challenges different EFs; participants observe specific cues on a frontal screen and concurrently are expected to execute well-coordinated movements. The video games included progressive levels of difficulty. Interestingly, cognitive benefits from exergaming increase with the dose of interactive mental challenge (Barcelos et al., 2015). To summarize, our exergame included a specific EF training under user-matched training levels that might trigger the beneficial effects on the measured EFs. On the other hand, balance exercise consisted of highly variable exercises, which in fact is an important and effective method to train motor learning (Healy et al., 2014), and might have, therefore, helped participants learn to adapt to new and changing situations, an important aspect for flexibility (Eslinger and Grattan, 1993). Finally, both interventions elicit beneficial effects on EFs while the exergame seems to be a more specific and efficient training method to improve EFs.

### Spatio-temporal gait parameters

No time × group interaction effects were present for any of the measured gait parameters. The balance group demonstrated significant within-group improvements of several gait parameters, however, merely in the single-task conditions. Balance training improves motor performance by changes on subcortical structures (Taube et al., 2008). Furthermore, the significant improvement for the walking speed parameter during fast and fast cognitive (dual-task) walking might indicate that improved balance abilities impact gait performance by changes of gait speed, especially during fast walking. Similarly, one recent study showed improved gait speed after balance training (Halvarsson et al., 2015). Furthermore, physical exercise positively influences dual-task walking by increasing the walking speed (Plummer et al., 2015). On the other hand, the exergame group showed significant within-group improvements for gait parameters under dual-task conditions. These significant positive within-group differences for gait parameters in the dual-task conditions confirm findings from previous pilot studies with similar results for dual-task related costs (de Bruin et al., 2011; Pichierri et al., 2012b). Exergame training combines enhancements of motor and cognitive functions that, therefore, positively influence gait performance under dual-task conditions (Pichierri et al., 2011, 2012a; Segev-Jacubovski et al., 2011). Moreover, divided attention is trained by concurrent observation of cognitive stimuli and performance of well-coordinated movements. Even more, participants were expected to observe the virtual environment for drifting symbols or figures and, at the same time, initiate steps on a pressure sensitive area. When using an outward step, participants needed to rapidly unload the leg they were falling toward to allow to take a step. This may be challenging from a cognitive, RT, and/or muscle power generation perspective (Egerton et al., 2011). The crucial point is that the exergame does not only require well-coordinated leg movements, but also requires cognitive work, e.g., sensing of stimuli, paying attention, and making quick decisions (de Bruin et al., 2010). Thus, a repeated practice of exergaming (dual-tasking), using task-specific training, might improve dual-task interference (Plummer et al., 2015). A transfer of the trained cognitive abilities on the concurrent performance of multiple tasks might happen (Bherer et al., 2005).

### Limitations

Some limitations of the study were the quite fit elderly participants, small sample size, and probably the short training intervention time. A modification of these factors might lead to interaction effects between the groups. Furthermore, the training time and intensity of the experimental and control group were not compatible because matching of the training content was not possible. Moreover, for the exergame training, each participant played the same amount of games, but no strict order of the game existed. For the EEG, two circumstances might limit the measurement: The naïve triggering approach and the use of a low-density EEG device.

## Conclusion

For aging humans, an exercise program that effectively addresses prefrontal brain activity and function, especially EFs and gait performance, might be important since age-associated reduction of EFs can impact gait and amplify the risk of falling (Mirelman et al., 2012). Further, two recent reviews, focusing on the interplay between physical functions and cognition, concluded that it seems important to combine motor and cognitive therapy into clinical practice to enable older adults to move safer in their physical environment (Segev-Jacubovski et al., 2011; Pichierri et al., 2012a). The present study illustrated that especially exergame training affects prefrontal theta RP and that exergame training and balance training positively influence EFs and gait performance to different extents. Thus, exergame training might be a promising future training strategy targeting prefrontal brain activity, EFs, and gait in elderly. Especially promising seems the effect that exergame is able to influence dual-task walking. Future studies should investigate cortical activity in additional brain areas during EF testing, i.e., using a high-density EEG device to substantiate the presented results. In addition, studies should focus on larger study sample sizes, longer intervention duration, and a strict training content for all the participants. Furthermore, the exergame (visual and acoustic) stimuli should be targeted on the specific EFs that have to be improved in elderly.

## Ethics statement

Ethics committee of the ETH Zurich, Switzerland (EK 2015-N-10). Before any measurements were performed, all eligible participants had to sign written informed consent according to the Declaration of Helsinki. The study involved no vulnerable populations.

## Author contributions

AS and RA developed the research question under the lead of EDB. The concept and design part was established by AS and RA while EDB acted as methodological council. AS and RA did data acquisition as well as, together with FG, analysis and interpretation of the results which was edited and improved by EDB. AS and RA produced an early version of the manuscript. FG and EDB substantially revised the manuscript to bring it to its current version. All authors have read and approved the final manuscript.

## Funding

This article was supported by the ETH Foundation through ETH Research Grant ETH-17 13-2.

### Conflict of interest statement

EDB was a co-founder of dividat, the spin-off company that developed the video games used in this study, and is associated to the company as an external advisor. No revenue was paid (or promised to be paid) directly to EDB or his institution over the 36 months prior to submission of the work. The rest of the authors AS, FG, and RA declare that the research was conducted in the absence of any commercial or financial relationships that could be construed as a potential conflict of interest. The reviewer RK and handling Editor declared their shared affiliation, and the handling Editor states that the process nevertheless met the standards of a fair and objective review.

## Abbreviations

EEG: electroencephalography
EF: executive function
FES-I: falls efficacy scale international
GDS: geriatric depression scale
MMSE: mini mental state examination
RP: relative power
RT: reaction time
TAP: test for attentional performance.
